# Emerging Mechanisms of Plant Responses to Abiotic Stress

**DOI:** 10.3390/plants14223445

**Published:** 2025-11-11

**Authors:** Wan Zhao, Xiaojie Chen, Jiahuan Wang, Zhongjie Cheng, Xuhui Ma, Qi Zheng, Zhaoshi Xu, Fuyan Zhang

**Affiliations:** 1 Henan Key Laboratory of Nuclear Agricultural Sciences, Isotope Institute Co., Ltd., Henan Academy of Sciences, Zhengzhou 450015, China; z_wendy523@hnas.ac.cn (W.Z.); czj0371@139.com (Z.C.);; 2 Institute of Chemistry, Henan Academy of Sciences, Zhengzhou 450002, China; 3 Hebei Seed Management General Station, Shijiazhuang 050031, China; 4 State Key Laboratory of Crop Gene Resources and Breeding, Institute of Crop Sciences, Chinese Academy of Agricultural Sciences (CAAS), Beijing 100081, China; 5 Joinhope Seed Co., Ltd., Changji 831000, China

**Keywords:** abiotic stress, signal transduction, transcriptional regulation, organellar communication, multi-stress tolerance

## Abstract

Plants continuously face multiple abiotic stresses, including drought, salinity, heat, cold, and heavy metal, that challenge cellular homeostasis and threaten global crop productivity. Recent research reveals that these stress responses are not isolated but interconnected through shared hormonal, redox, and transcriptional networks. This review provides an integrative synthesis of current advances in stress signaling, emphasizing how perception, transduction, and memory layers are hierarchically organized across distinct stress types. We outline key regulatory hubs—such as ABA-centered hormonal crosstalk, chloroplast-nucleus redox communication, and epigenetic priming—that coordinate systemic tolerance. Furthermore, we highlight emerging evidence for stress-specific modules that operate under combined stresses (e.g., drought–heat, salinity–cold), providing a unified framework for understanding how plants integrate multi-dimensional signals. This synthesis offers a conceptual perspective linking signaling architecture to adaptive outcomes, aiming to inform future strategies for engineering multi-stress-resilient crops.

## 1. Introduction

Plants, being sessile organisms, are constantly exposed to a wide array of abiotic stresses—including drought, salinity, extreme temperatures (heat and cold), and oxidative stress—which jeopardize their survival, growth, and productivity. These environmental constraints have become increasingly severe and unpredictable under global climate change, posing significant challenges to food security and the sustainability of agricultural systems [1,2].

While each type of abiotic stress triggers distinct physiological and molecular responses, accumulating evidence indicates that plants employ highly interconnected signaling networks to enable both specific and integrated responses across stress conditions [3,4,5].

At the molecular level, stress responses are coordinated through rapid sensing mechanisms and signal transduction pathways involving secondary messengers such as calcium ions (Ca^2+^), reactive oxygen species (ROS), and phytohormones including abscisic acid (ABA), ethylene, salicylic acid (SA), jasmonates (JAs), gibberellins (GAs), and brassinosteroids (BRs) [6,7,8,9,10]. These signals activate transcriptional cascades driven by diverse transcription factor families (e.g., MYB, bZIP, NAC, and DREB/CBF), which in turn regulate stress-responsive genes to mount protective responses [11,12,13,14].

In addition to transcriptional regulation, cells employ fine-tuning at the post-transcriptional, translational, and post-translational levels to enable dynamic and reversible adaptation to stress [15,16,17]. Moreover, epigenetic mechanisms and organelle-to-nucleus signaling have emerged as critical regulators of stress memory and cross-organellar coordination [18,19,20,21].

Recent studies have revealed novel insights into how plants perceive and integrate environmental signals. These include the discovery of multisensor thermosensory systems, ROS waves for systemic signaling, chloroplast-derived retrograde pathways, and membrane-associated ion perception complexes [20,21,22,23,24]. Cutting-edge technologies—such as chromatin conformation capture, CRISPR-based gene editing, single-cell transcriptomics, and live-cell imaging—have dramatically advanced our understanding of stress signaling in spatial and temporal dimensions [25,26,27,28]. Despite these breakthroughs, a key unresolved challenge remains: how to confer stress tolerance without penalizing growth. Understanding the mechanisms that decouple growth inhibition from defense activation is critical for breeding climate-resilient crops. For instance, strategies such as tissue-specific hormone signaling or translation-preserving mechanisms have shown promise in bypassing the traditional growth–defense trade-off [21,29].

Despite extensive progress in dissecting these mechanisms, our understanding of how plants integrate multiple stress cues and maintain long-term adaptation through molecular and epigenetic memory remains limited.

To address these gaps, this review integrates molecular signaling, transcriptional regulation, and epigenetic memory into a unified framework of abiotic stress adaptation. We systematically summarize emerging mechanisms underlying plant responses to drought, salinity, heat, cold, and heavy metal stresses. This synthesis provides a comprehensive overview of dynamic, multilayered defense systems in plants and highlights future directions for developing resilient crops under increasingly complex environmental conditions.

## 2. Emerging Mechanisms Underlying Plant Responses to Major Abiotic Stresses

### 2.1. Drought Stress Responses

Drought stands as one of the most severe environmental constraints impacting plant growth and agricultural productivity. It triggers a multifaceted response encompassing early sensing mechanisms, extensive signal transduction networks, and diverse physiological and molecular adaptations [30,31]. Plants must optimize water use efficiency and mitigate cellular damage while maintaining growth to the greatest extent possible. This section outlines key molecular pathways and adaptive strategies that enable plants to respond effectively to drought stress, beginning with the pivotal role of ABA signaling. In line with recent advances, we emphasize that drought acclimation integrates an ABA core with Ca^2+^-dependent feedback, hormone crosstalk, and long-distance ROS-Ca^2+^ waves, and is further shaped by microbiome- and epigenetics-driven plasticity [19,30,32,33].

#### 2.1.1. ABA Signaling: A Central Hormonal Regulator

**Figure 1:**
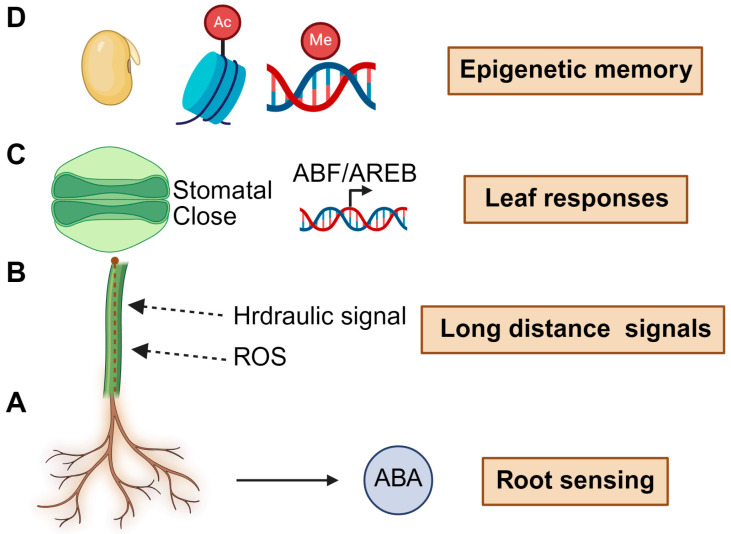
Integrated mechanisms of plant drought response: from root sensing to epigenetic memory. Drought stress triggers a multilayered response in plants that spans environmental sensing, systemic signaling, physiological adaptation, and long-term memory formation. (**A**) At the root level, cells detect declining soil water potential and initiate the biosynthesis of ABA. (**B**) These drought cues are then transmitted to aerial tissues via hydraulic signals and ROS traveling through the xylem. (**C**) In the leaves, ABA signaling induces stomatal closure by modulating ion channels and activates key drought-responsive transcription factors such as ABF/AREB. (**D**) At the chromatin level, epigenetic modifications-including histone acetylation and DNA methylation at stress-inducible loci-establish a form of drought memory, enabling plants to respond more rapidly and robustly upon re-exposure to drought, and in some cases, confer transgenerational resilience.

Drought stress initiates a complex network of sensory, signaling, and physiological responses in plants that collectively promote water conservation and survival. A central regulator of drought response is the phytohormone ABA, which accumulates in response to decreasing soil water potential and orchestrates a broad array of downstream defense mechanisms [3,34,35] (Figure 1A). In guard cells, ABA triggers stomatal closure to reduce transpiration, primarily through the activation of SnRK2.6/OST1, which phosphorylates ion channels such as SLAC1, inducing anion efflux and subsequent turgor loss [36]. The conservation of this pathway across species has facilitated its targeted manipulation in crops such as rice (*Oryza sativa*) and maize (*Zea mays*), resulting in enhanced drought resilience [27]. Beyond the canonical PYR/PYL/RCAR receptors, recent studies have identified NRT1.1B, a dual-function nitrate transporter, as an additional ABA receptor that integrates nitrate signaling with ABA-mediated stress responses [37]. This discovery expands the paradigm of ABA perception and highlights the tight interplay between hormone signaling and nutrient availability. Consistently, stress-induced microRNAs-such as miR393-TIR1/AFB and miR169-NF-YA-buffer growth–defense trade-offs and fine-tune ABA-linked transcription under drought and salinity [38]. Recent studies suggest ABA perception is not limited to canonical receptors, shifting our understanding from a single hormone pathway to nutrient-hormone integrated sensing. Beyond endogenous hormones, microbiome signals can potentiate ABA programs to remodel root architecture and epigenetic states under water deficit, thereby enhancing drought tolerance [19].

#### 2.1.2. Hormonal Crosstalk Among ABA, JA, and SA Under Drought Stress

In addition to abscisic acid (ABA), other phytohormones such as jasmonates (JAs) and salicylic acid (SA) actively participate in drought response and adaptation. JAs accumulate rapidly during dehydration and regulate stomatal closure, reactive oxygen species (ROS) detoxification, and the expression of stress-inducible genes via the COI1-JAZ-MYC2 module. JAs often act synergistically with ABA to reinforce osmotic adjustment and defense activation [39]. Conversely, SA can function either antagonistically or cooperatively with ABA depending on stress intensity and duration; SA-induced activation of NPR1 and TGA transcription factors contributes to antioxidant capacity and stress recovery [40]. The dynamic ABA-JA-SA crosstalk thus coordinates the growth–defense balance by integrating hormonal, redox, and transcriptional signaling, providing plants with a flexible strategy to optimize drought resilience. This highlights a conceptual shift from “single-hormone control” toward dynamic hormone network computation of stress intensity and duration.

#### 2.1.3. Brassinosteroid Signaling and the Growth–Defense Trade-Off

In parallel with ABA-JA-SA interactions, brassinosteroids contribute a complementary layer that reconciles stress tolerance with growth maintenance. Brassinosteroids (BRs), a class of growth-regulating hormones, have also been implicated in drought adaptation. Overexpression of *BRI1*, a ubiquitous BR receptor, enhances drought tolerance but compromises growth due to reduced stomatal density [29]. In contrast, tissue-specific expression of BRL3, a BR receptor predominantly expressed in vascular tissue, confers drought tolerance without negatively affecting growth. Plants overexpressing *BRL3* exhibit elevated levels of osmoprotectants and activate stress-responsive genes specifically in vascular cells. These observations suggest that spatially confined BR signaling enables systemic metabolic adjustment while maintaining growth performance, offering a promising strategy to decouple the trade-offs between growth and defense in crop improvement. Recent multi-omic studies further indicate that growth–defense balance is encoded at cell-type resolution, arguing for tissue-targeted hormone engineering rather than bulk overexpression [41]. Thus, BR biology under drought now serves as a model for spatial rewiring of hormone perception to avoid classical growth penalties.

#### 2.1.4. Hydraulic and Electrical Signaling

In addition to hormonal signaling, plants perceive drought via hydraulic and electrical signals transmitted from roots to shoots (Figure 1B). As soil dries, reduced turgor in root cells induces changes in xylem tension, functioning as rapid hydraulic signals that can precede ABA accumulation in leaves [35,42,43,44]. Furthermore, drought triggers systemic ROS and Ca^2+^ waves that act as mobile stress signals [21] (Figure 1B). Analogous to high-light stress responses, ROS generated by the NADPH oxidase RBOHD initiate cell-to-cell signaling via plasmodesmata-facilitated by PDLP1 and PDLP5-and propagate through ROS-Ca^2+^ feedback loops to systemically activate defense genes [45]. These findings indicate that drought sensing is not exclusively hormonal but involves multimodal signaling, including biophysical and redox-based pathways. The emerging conceptual advance here is that hydraulic/electrical waves are not passive by-products but active, instructive signals that “pre-prime” distal tissues before hormonal accumulation.

#### 2.1.5. Root System Plasticity and Water Acquisition

Morphological adaptations under drought conditions, particularly root system plasticity, play a crucial role in enhancing water acquisition. Moderate drought stress often promotes root elongation and modifies root angle to facilitate exploration of deeper soil layers [46]. For example, an ABA-auxin signaling module has been identified as a key regulator of root gravitropism and root-to-shoot biomass ratio under water-deficient conditions [9]. Additionally, loss-of-function mutants of the NAC transcription factor *XND1* demonstrate increased root hydraulic conductivity through upregulation of aquaporin activity, thereby improving shoot hydration and biomass accumulation under drought [47]. Overexpression of specific aquaporins, such as PIP-type aquaporins also enhances root water transport capacity [48,49]. However, inconsistencies between mRNA expression levels and observed drought responses suggest that post-translational regulation of aquaporins plays a pivotal role [50]. Cell-type-resolved ‘same-cell’ multiome maps under osmotic stress show that root developmental trajectories and chromatin accessibility states are jointly rewired, providing a mechanistic basis for plastic root foraging in drying soils [41]. Conceptually, root plasticity is increasingly viewed less as “morphological change” and more as a regulatory output of hormone-mechanics integration tuned for water foraging strategy.

#### 2.1.6. Transcriptional Regulation and Epigenetic Reprogramming

Sustained adaptation requires transcriptional reprogramming and chromatin remodeling to stabilize drought-responsive states. At the transcriptional level, ABA-responsive bZIP transcription factors such as ABF/AREB directly bind to drought-inducible promoters and regulate gene expression (Figure 1C). A notable advance involved the use of CRISPR activation (CRISPRa) to epigenetically enhance expression of *AREB1* (*ABF2*) using a dCas9-histone acetyltransferase fusion targeted to the *AREB1* promoter [27]. This approach conferred drought tolerance in *Arabidopsis* with minimal growth penalties, highlighting the potential of targeted epigenetic modulation over constitutive overexpression for improving drought resilience. This exemplifies the conceptual transition from descriptive transcriptomics toward causal, programmable control of drought transcriptional states.

#### 2.1.7. Drought Memory and Transgenerational Priming

Emerging evidence highlights the significance of drought memory and epigenetic regulation in enhancing plant resilience (Figure 1D). Plants previously exposed to drought stress often exhibit a more robust response upon subsequent exposure, a phenomenon known as drought priming. In *Brassica napus*, progeny of drought-experienced parents display enhanced seedling vigor under water-limited conditions, which associated with altered seed composition and epigenetic marks in embryos [51,52] (Figure 1D). Intra-generational drought memory has also been linked to histone modifications, DNA methylation, and 3D chromatin remodeling at stress-responsive loci, as shown by Hi-C and chromatin conformation analyses [25,30,53,54]. These chromatin-level changes may maintain stress-responsive genes in a transcriptionally poised state, facilitating rapid reactivation upon re-exposure to drought stress. Conceptually, drought memory represents a shift from “momentary stress response” to dynamic state-storage that can persist across cell lineages and even generations.

Together, these findings illustrate that plant drought adaptation is governed by an integrated signaling network involving hormonal, hydraulic, electrical, redox, and epigenetic regulatory mechanisms. Layering the ABA core with Ca^2+^ feedback (CBL1/9-CIPK1), microbiome-coupled ABA reprogramming, compartmentalized AsA-GSH control of ROS, and cell-type-resolved regulatory trajectories provides an updated, mechanism-rich account of drought acclimation [33,41]. A deeper understanding and precise manipulating these regulatory layers-particularly with precision tools like CRISPRa and tissue-specific signaling modulation-present promising opportunities for engineering drought-resilient crops without compromising growth performance.

### 2.2. Salinity Stress Responses

Salinity represents one of the most widespread abiotic stresses that significantly impair global crop productivity, particularly in arid and semi-arid regions. Elevated soil salinity imposes both osmotic and ionic stress on plants, leading to impaired water uptake, disruption of nutrient homeostasis, and compromised cellular homeostasis [2,55,56]. Although certain signaling components are shared with drought responses, plants have also evolved distinct salinity-specific mechanisms that regulate ion transport, compartmentalization, and specialized metabolic pathways. Recent comparative synthesis confirms that salinity-triggered responses span sensing, ion transport, osmotic adjustment, and oxidative signaling, while also showing species- and tissue-specific regulatory repertoires [57]. A comprehensive understanding of these responses is essential for the development of salt-tolerant crops within saline-prone agricultural systems.

Conceptual synthesis note: such mechanistic comparisons also highlight that halophytes rewire core modules (e.g., SOS targeting, osmolyte metabolism, antioxidant buffering) in qualitatively different ways than glycophytes, indicating that salt tolerance is not a linear enhancement of known pathways but a regulatory reallocation and rewiring of basal programs.

#### 2.2.1. Ionic Homeostasis and the SOS Pathway

**Figure 2:**
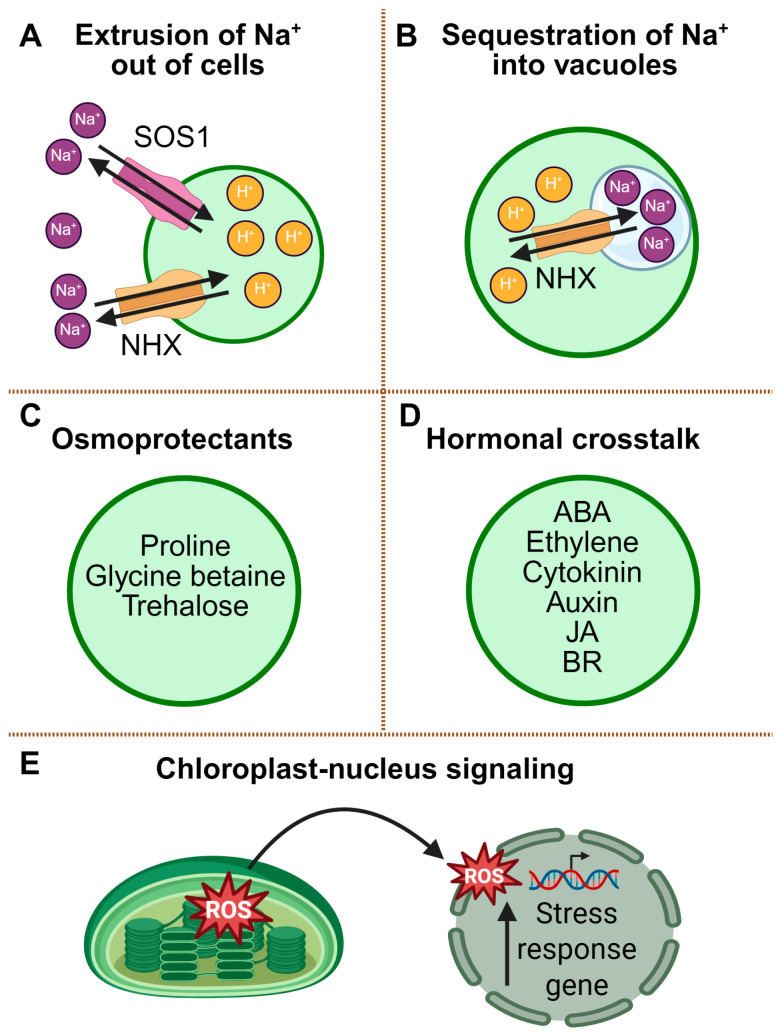
Salinity stress responses in plants. Key physiological and molecular mechanisms involved in plant adaptation to high salinity stress are shown. (**A**) Na^+^ extrusion is mediated by plasma membrane Na^+^/H^+^ antiporters (e.g., SOS1), which remove excess Na^+^ from the cytosol. (**B**) Vacuolar sequestration of Na^+^ via NHX-type Na^+^/H^+^ antiporters contributes to maintaining a favorable cytosolic K^+^/Na^+^ ratio. (**C**) Accumulation of compatible solutes such as proline, glycine betaine, and trehalose helps stabilize proteins and maintain osmotic balance. (**D**) Hormonal regulation involving ABA, ethylene, cytokinin, auxin, and jasmonic acid (JA) and brassinosteroid (BR) modulates stress signaling and plant development under saline conditions. (**E**) Salt stress induces the production of ROS in chloroplasts, which activate the expression of stress-responsive genes in the nucleus.

A central aspect of salinity tolerance lies in the maintenance of ionic homeostasis, with a particular emphasis on regulating intracellular Na^+^ levels. The Salt Overly Sensitive (SOS) signaling pathway stands as a paradigmatic example of this strategy and has served as a foundational model for elucidating the regulation of ion transport under salt stress conditions. In *Arabidopsis*, *SOS1* encodes a plasma membrane-localized Na^+^/H^+^ antiporter, whereas SOS2 (CIPK24) and SOS3 (CBL4) form a calcium-dependent kinase complex that activates SOS1 under salt stress [58] (Figure 2A). Recent cryo-electron microscopy studies have demonstrated that SOS1 functions as a homodimer, which undergoes conformational shifts between its inactive and phosphorylated active states [59,60]. These structural insights explain how phosphorylation of SOS1 by SOS2 enhances its Na^+^ extrusion capacity. This demonstrates that the SOS module functions not merely as an ion export system but as a regulatory hub whose structural plasticity enables programmable Na^+^ homeostasis under fluctuating salinity conditions.

Notably, a homolog of SOS1 (SbiSOS1) in the halophytic species *Salicornia bigelovii* was recently found to localize not to the plasma membrane but to the tonoplast [61]. In this species, SbiSOS1 acts as a vacuolar Na^+^/H^+^ antiporter, mediating the transport of Na^+^ into the vacuole instead of exporting it from the cell. This relocalization helps prevents cytosolic Na^+^ accumulation by compartmentalizing salt into a safer intracellular compartment, employing the same antiporter mechanism at a different membrane. Although vacuolar Na^+^ sequestration is typically mediated by NHX-type tonoplast antiporters [62], *Salicornia* appears to have co-opted the SOS1 system to perform a similar role under extreme salinity (Figure 2B). This example highlights that the subcellular targeting of transport proteins—via differential signal peptides or membrane insertion motifs—can be as critical as their catalytic activity in the evolution of stress adaptation mechanisms.

#### 2.2.2. Osmotic Adjustment and Compatible Solute Accumulation

Beyond cytosolic Na^+^ extrusion and homeostasis, plants must also restore osmotic balance to preserve cellular function under salinity. To counteract osmotic imbalance induced by salinity, plants accumulate compatible solutes such as proline, glycine betaine, and trehalose, which contribute to the stabilization of proteins and cellular structures under salt stress (Figure 2C). These osmolytes not only improve water retention but also protect the photosynthetic machinery and enzymatic systems from ionic and oxidative damage. In tomato, exposure to combined heat and salt stress resulted in elevated levels of glycine betaine and trehalose, leading to enhanced osmotic adjustment and sustained photosynthetic performance compared to salt stress alone [56]. Furthermore, improved K^+^ retention and a reduced Na^+^/K^+^ ratio in stressed tissues suggest a synergy between ion homeostasis and osmolyte function [32]. Thus, osmolyte reprogramming should be viewed not as an additive protective layer but as a dynamic metabolic switch that actively couples osmosis, redox buffering, and ion partitioning under saline stress.

#### 2.2.3. Hormonal Crosstalk

These biophysical adjustments are integrated by hormonal networks that coordinate organ-level growth and stress responses. Hormone interactions play a pivotal role in fine-tuning plant responses to salinity. Abscisic acid (ABA) accumulates rapidly under salt stress and promotes stomatal closure as well as the expression of stress-responsive genes. ABA-deficient mutants exhibit hypersensitivity to salinity, underscoring its protective function [63]. Ethylene acts antagonistically to ABA, particularly during seed germination; elevated ABA levels induce dormancy under salinity, whereas ethylene can mitigate this inhibition [13,63,64]. Salinity also suppresses cytokinin and auxin signaling, resulting in reduced shoot growth and increased root system plasticity [55]. Conversely, brassinosteroids (BRs) and jasmonic acid (JA) positively regulate salt tolerance by enhancing antioxidant capacity and modulating the expression of ion transporters through transcription factors such as the nuclear factor Y (NF-Y) complex [65] (Figure 2D). This hormonal cross-regulation indicates that salt tolerance is not determined by ABA dominance alone but by context-dependent multi-hormone logic that reallocates growth and metabolic resources.

#### 2.2.4. ROS Signaling and Chloroplast-to-Nucleus Communication

In parallel, redox signals arising from organelles relay salinity stress to the nucleus to reprogram gene expression. High salinity leads to the accumulation of ROS within chloroplasts, mitochondria, and peroxisomes, which poses a risk of oxidative damage if not tightly controlled. However, spatially and temporally regulated ROS production also plays a critical signaling role (Figure 2E). A notable example is the chloroplast-to-nucleus retrograde signaling pathway in *Arabidopsis*, mediated by the protease EGY3 and the Cu/Zn superoxide dismutase CSD2 [20] (Figure 2E). Under salt stress, this protein complex facilitates the release of H_2_O_2_ from chloroplasts, which subsequently translocates to the nucleus and modulates gene expression patterns to promote stress tolerance. This mechanism illustrates the importance of organelle-nucleus communication in integrating redox signals into the plant’s broader stress response network. In non-stress conditions, PP2C.D6/D7 directly suppress SOS1 Na^+^/H^+^ exchange activity, while under salinity this brake is released by SCaBP8/CBL10, providing a context-dependent switch for SOS1 activation [66]. These findings support a view that organelle ROS waves do not simply report oxidative status but function as real-time retrograde encoders that rewire nuclear programs under salinity.

#### 2.2.5. Metabolic Reprogramming

The transcriptional outputs of redox signaling are accompanied by broad metabolic rewiring to fuel ion transport and osmolyte biosynthesis. Salt stress induces profound shifts in primary metabolism. Metabolomic analyses have revealed marked changes in the levels of tricarboxylic acid (TCA) cycle intermediates, sugars, and amino acids such as γ-aminobutyric acid (GABA) under salinity conditions [67,68]. These metabolic adjustments facilitate energy production, osmotic homeostasis, and reactive oxygen species (ROS) scavenging. In salt-tolerant genotypes, enhanced respiration and glycolytic activities compensate for reduced photosynthesis efficiency, thereby supplying energy for active Na^+^ transport and osmolyte biosynthesis. Therefore, metabolic rewiring represents an active strategy to maintain bioenergetic flexibility rather than a passive metabolic consequence of stress.

#### 2.2.6. Translational Protection

In extremophytes, additional protective layers operate at the translational level to stabilize protein synthesis under salt stress. Beyond canonical signaling pathways, recent studies on extremophytes have uncovered new protective layers. In *Salicornia*, a salt-induced protein named SALTY, containing an RGG domain, was identified to localize to the endoplasmic reticulum where it associates with ribosomes and RNA [61]. SALTY is hypothesized to stabilize protein synthesis under salt stress, and heterologous expression in yeast conferred enhanced salt tolerance, suggesting a conserved protective potential. This highlights translational homeostasis as an overlooked yet essential aspect of plant adaptation to salt stress. These discoveries collectively reveal that regulation of translation is not simply a downstream bottleneck but a direct determinant of salinity resilience.

### 2.3. Heat Stress Responses

Global warming and the increasing frequency of extreme high-temperature events have elevated heat stress to a critical challenge for plant development and agricultural productivity [69]. Elevated temperatures can disrupt protein folding, compromise membrane stability, impair photosynthetic efficiency, and induce excessive accumulation of reactive oxygen species (ROS). To mitigate these adverse effects, plants have evolved sophisticated strategies spanning multiple regulatory levels, including thermosensing, transcriptional reprogramming, translation control, and epigenetic memory formation [15,18,22]. This section outlines recent advances in understanding plant responses to heat stress, with a focus on elucidating the mechanisms by which plants perceive temperature fluctuations and initiate adaptive responses to maintain growth and ensure survival under heat stress conditions. In addition, we also took into account recent studies applying heat stress in agronomic contexts, such as the 2025 *Ecotoxicology* report showing how heat stress interacts with microplastics to reshape ROS production and antioxidant responses in wheat and maize [70].

#### 2.3.1. Thermosensing via Multisensor Systems

**Figure 3:**
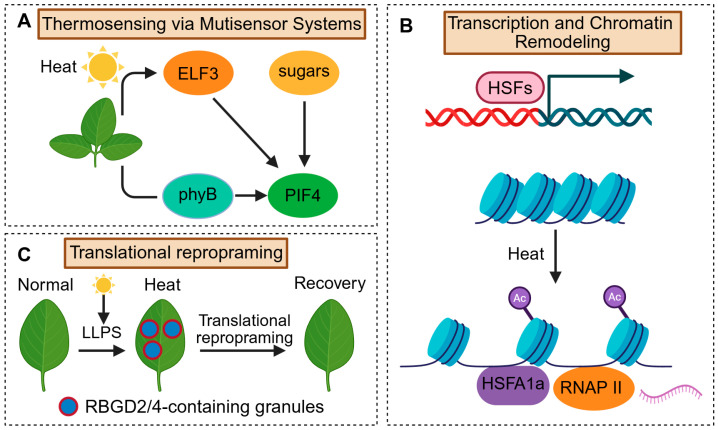
Multilayered mechanisms of plant heat stress responses. (**A**) Heat stress is sensed via multiple systems including phyB, ELF3, and sugar signals, all converging on PIF4 to drive thermomorphogenesis. (**B**) Heat activates HSFs such as HSFA1a, which induce heat-responsive gene expression and mediate chromatin remodeling. (**C**) Under heat, RBGD2/4 undergo LLPS to form stress granules, enabling translational reprogramming and promoting plant survival.

Thermosensory pathways ultimately activate heat-shock transcription factors, triggering genome-wide induction of protective genes. Heat stress, particularly during acute high-temperature events such as heatwaves, represents a severe threat to plant growth and yield. In response, plants have evolved both rapid protective mechanisms and longer-term morphological adaptations, collectively termed thermomorphogenesis. These include hypocotyl/stem elongation, leaf hyponasty, and modifications in flowering time under prolonged exposure to elevated temperatures [22,71,72,73,74]. One well-established thermosensory pathway involves the red/far-red photoreceptor phytochrome B (phyB), which acts as a molecular thermometer (Figure 3A). In its biologically active Pfr conformation, phyB binds to PHYTOCHROME INTERACTING FACTOR 4 (PIF4) and facilitates its degradation-with PIF4 serving as a central regulator of thermomorphogenesis. Elevated temperatures accelerate the thermal reversion of phyB to its inactive Pr conformation, which in turn stabilizes PIF4 and triggers the downstream transcription of growth-related genes-including those encoding components of auxin biosynthesis pathways [74,75,76,77]. Notably, phyB-mediated temperature sensing exhibits maximal efficacy under shade or nighttime conditions. By contrast, under daylight conditions, sustained light signals maintain phyB in its active (Pfr) form, thus constraining its ability to sense temperature [78]. Daytime thermomorphogenesis is driven by a multisensor framework integrating phyB inactivation, PIF4 stabilization, and ELF3 relief, which together tune growth under warm light conditions [79]. Under heat, miRNA circuits (e.g., miR156-SPL and miR398-CSD/CCS) reshape thermotolerance and recovery by coupling developmental state with redox capacity and HSF/HSP programs [80,81,82]. While phyB-PIF4-driven thermomorphogenesis is well established in *Arabidopsis*, its quantitative contribution varies among species and growth conditions, indicating a context-dependent module layered upon more conserved heat-protective programs.

Recent studies have elucidated additional thermosensory mechanisms that compensate for phyB’s reduced activity under strong light. High temperature induces chloroplast starch degradation and sucrose accumulation, which stabilizes PIF4 protein, likely via sugar-derived signals such as trehalose-6-phosphate [79]. Concurrently, elevated temperatures trigger liquid–liquid phase separation of the evening complex component ELF3, a transcriptional repressor of PIF4. The formation of ELF3 condensates impairs its DNA-binding ability, thereby relieving repression on PIF4 transcription [83,84]. Collectively, these phyB, sugar, and ELF3-mediated mechanisms converge on PIF4 to finely tune thermomorphogenic growth (Figure 3A).

#### 2.3.2. Additional Thermosensory Components and Signaling Pathways

Beyond phyB and ELF3, plants employ a diverse array of additional thermosensory systems. Under heat stress, alterations in membrane fluidity can activate calcium influx via mechanosensitive channels, leading to Ca^2+^ transients that initiate downstream signaling cascades. Chromatin-level thermosensors have also been hypothesized-nucleosomes containing the histone variant H2A.Z exhibit reduced stability at elevated temperatures, which, may facilitate promoter accessibility and transcription initiation at thermoresponsive genomic loci [18,85,86]. Emerging evidence, however, supports a distributed thermosensory network-rather than a single master sensor [6] that encompasses photoreceptors, chromatin components, metabolic cues, and membrane dynamics. Thus, we consider membrane/Ca^2+^ and redox cues as core features across taxa, with photoreceptor- and clock-linked inputs tuning species-specific growth outcomes. Thermosensing reflects a distributed system integrating chromatin, membranes, metabolites, and light.

#### 2.3.3. Heat-Induced Transcriptional Regulation and Chromatin Remodeling

To counteract acute thermal damage, plants activate the canonical heat shock response (HSR), characterized by the robust induction of heat shock proteins (HSPs). These HSPs act as molecular chaperones to refold misfolded proteins. The transcriptional regulation of HSR is governed by heat shock transcription factors (HSFs), particularly members of the HSFA1 family, such as HSFA1a in *Arabidopsis thaliana* and tomato (Figure 3B). Beyond their classical role in inducing target genes, HSFA1s also mediate the reorganization of genome architecture during heat stress. Hi-C analyses in tomato, for instance, demonstrate that HSFA1a facilitates the widespread formation of promoter-enhancer loop under heat conditions, thereby coordinating the activation of heat-responsive gene clusters [26]. Loss of HSFA1a disrupts these chromatin interactions and impairs transcriptional responsiveness, underscoring its dual function as both a transcriptional activator and a chromatin organizer (Figure 3B). HSFs act not only as transcription factors but as 3D genome organizers under heat.

#### 2.3.4. Translational Reprogramming and Post-Transcriptional Control

Transcriptional activation is complemented by translational reprogramming that reallocates ribosomes toward heat-adaptive mRNAs. Recent studies reveal that translational regulation constitutes a critical regulatory layer for heat stress adaptation. During heat stress episodes, global protein synthesis is transiently suppressed, whereas the selective translation of key regulatory factors is either maintained or upregulated. For example, the mRNA of *PIF7* contains a thermoresponsive hairpin structure in its 5′-UTR, which unfolds at elevated temperatures (~28 °C) to promote the translation of PIF7 itself [87]. Similarly, transcripts of heat shock proteins (HSPs) possess structural or sequence features that support their preferential translation under thermal stress [88]. In parallel, RNA-binding proteins (RBPs) such as RBGD2 and RBGD4 undergo phase separation to form stress granules. These granules temporarily sequester non-essential mRNAs, thereby enabling stress-prioritized translational programs [89,90]. At the translation layer, intermittent heat elicits priming-dependent restoration of translational capacity and co-translational mRNA-decay control, underscoring a post-transcriptional dimension of thermomemory [91]. Collectively, these mechanisms ensure efficient cellular resource allocation by maintaining the synthesis of stress-adaptive essential proteins and concurrently conserving energy under conditions of heat stress (Figure 3C). Transcriptional memory after heat involves HSFA2-dependent recruitment of the Mediator kinase module to memory loci, enhancing Pol II activity and enabling hyper-induction upon re-exposure [92], while the field now frames thermomemory as a dynamic balance between maintenance and resetting [93]. Translation actively reprioritizes proteome allocation during heat, independent of transcription.

#### 2.3.5. Heat Memory and Acclimation Mechanisms

These acute responses consolidate into heat memory, enabling faster and stronger reactivation upon recurrent stress. Plants exhibit the capacity to acquire heat stress memory, whereby prior exposure to moderate heat (e.g., 37 °C) improves their survival under subsequent, more severe thermal stress (e.g., 45 °C). Such acclimation relies on the sustained accumulation of small heat shock proteins (sHSPs) and transcription factors in a ‘primed’ state. Chromatin-level modifications also contribute to transcriptional memory-H3K4 demethylation dynamics at heat-responsive gene loci facilitate the rapid reactivation of these loci upon repeated stress exposure [15]. These molecular memory mechanisms collectively provide a fitness benefit by promoting faster and more potent activation of defensive responses during recurrent stress. Understanding how these mechanisms are regulated is key to developing or engineering crops varieties with enhanced thermotolerance.

### 2.4. Cold Stress Responses

Low temperature is another major abiotic stress that limits plant growth, development, and geographic distribution. Unlike heat stress, which rapidly denatures proteins and disrupts membrane integrity, cold stress gradually impairs physiological functions by reducing enzymatic activity, increasing membrane rigidity, and-under freezing conditions-inducing intracellular ice formation [94]. To cope with seasonal and diurnal temperature fluctuations, plants have evolved diverse adaptive strategies, including cold acclimation, the activation of cold-responsive genes, and metabolic adjustments [16,95,96]. This section explores the molecular and physiological mechanisms underlying plant responses to cold, focusing on recent insights into cold sensing, transcriptional regulation, redox signaling, and inter-organellar communication.

#### 2.4.1. Cold Perception and Calcium Signaling

**Figure 4:**
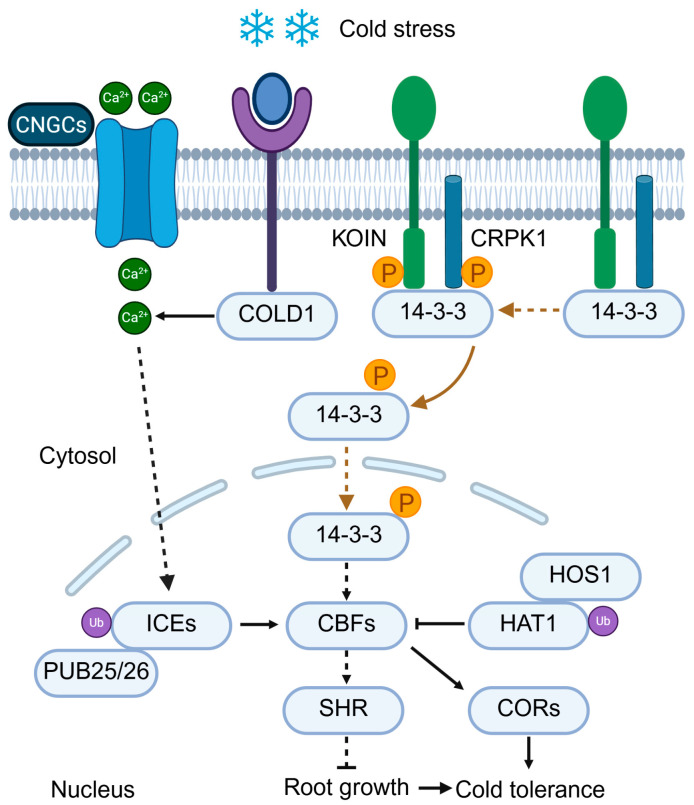
Cold stress signaling in plants. Cold stress is sensed by COLD1 and candidate channels such as CNGCs, triggering cytosolic Ca^2+^ influx that activates the ICE1-CBF-COR pathway. In parallel, the receptor-like kinase module KOIN/CRPK1 phosphorylates 14-3-3 proteins, which translocate into the nucleus to regulate CBFs. ICE1 stability is dynamically controlled by PUB25/26 via K63-linked ubiquitination for stabilization during early stress and K48-linked ubiquitination for degradation at later stages. HOS1 further promotes ICE1 degradation under warm conditions, while HAT1 represses CORs but is degraded upon cold exposure. Activated CBFs induce COR genes to enhance cold tolerance and regulate SHR to balance root growth under stress.

Cold stress, encompassing both chilling (low non-freezing temperatures) and freezing conditions, negatively affects plant physiology by reducing membrane fluidity, impairing enzymatic activity, and promoting the formation of intracellular ice crystals. Plants native to temperate regions can undergo cold acclimation-a process in which exposure to non-freezing low temperatures induces physiological adaptations that enhance freezing tolerance. These adaptations include the accumulation of cryoprotectants (e.g., soluble sugars), antifreeze proteins, and alterations in membrane lipid composition [14,97]. Although the precise mechanism of cold sensing remains incompletely understood, early cellular responses involve changes in membrane rigidity and cytoskeletal rearrangement, which trigger calcium influx. In rice, the *COLD1* gene encodes a protein localized to the plasma membrane and endoplasmic reticulum that interacts with the G-protein α subunit RGA1 to perceive chilling stress and mediate Ca^2+^ influx, thereby conferring chilling tolerance and accounting for the subspecies-specific differences in cold adaptability [98] (Figure 4). Whether COLD1-like sensing operates broadly across species remains unclear.

Cold-induced cytosolic Ca^2+^ bursts are believed to originate from mechanosensitive and cold-sensitive ion channels. Candidate sensors include cyclic nucleotide-gated channels (CNGCs), which facilitate Ca^2+^ influx [17,99] (Figure 4). The calcium signal is subsequently decoded by calcium-binding proteins, such as calmodulin and calcineurin B-like proteins (CBLs), which activate downstream kinases including Open Stomata 1 (OST1) and calmodulin kinase II (CAMKII)-like proteins, thus propagating cold-responsive signaling cascades.

#### 2.4.2. The ICE1-CBF-COR Module

A key regulatory module in cold acclimation is the CBF (C-repeat binding factor) pathway, also known as the DREB1 pathway (Figure 4). Upon cold exposure, CBF transcription factors are rapidly upregulated and induce the expression of over 100 cold-responsive (COR) genes involved in osmoprotection, dehydration resistance, and membrane stabilization [14,97,100]. The primary upstream regulator of CBFs is ICE1 (Inducer of CBF Expression 1), a constitutively nuclear basic helix-loop-helix (bHLH) transcription factor. Under warm conditions, ICE1 is ubiquitinated by the E3 ligase HOS1 (High expression of osmotically responsive genes 1) and subsequently degraded [101]. Upon cold exposure, HOS1 activity is suppressed, while ICE1 undergoes stabilizing post-translational modifications, including phosphorylation by OST1 and SUMOylation, enhancing its DNA-binding activity and nuclear stability [102,103] (Figure 4).

In *Arabidopsis*, two U-box E3 ligases, PUB25 and PUB26, dynamically regulate ICE1 stability via differential ubiquitination during cold stress. Specifically, during the early stages of cold stress, PUB25/26 promotes K63-linked ubiquitination of ICE1, thereby stabilizing ICE1 and maximizing the expression of CBF. In contrast, prolonged cold exposure induces K48-linked ubiquitination of ICE1, which in turn leads to the degradation of ICE1 and the attenuation of the cold response [104] (Figure 4). This regulatory axis enables the rapid activation of CBF3 and its homologs within hours of cold stress onset. Notably, OST1-previously characterized in ABA signaling-links drought and cold stress responses by modulating the activity of ICE1. Beyond cold acclimation, CBF3 has also been demonstrated to play a crucial role in root development under cold stress: it maintains the identity of the root stem cell niche and meristem activity, thereby coupling stress adaptation with growth regulation [105]. Recent studies further revealed that a plasma membrane-localized RLK-RLCK module, comprising KOIN and CRPK1, modulates cold-induced root growth inhibition via the 14-3-3-CBF-SHR signaling axis. This module links the activity of CBF3 to root meristem regulation under cold stress [106] (Figure 4). ICE1-CBF functions as a dynamic switch with time-phased ubiquitination logic, not a static pathway.

#### 2.4.3. Redox Regulation of CBF Activity

In addition to transcriptional control, CBF proteins undergo redox-dependent regulation. Recent studies have revealed that CBFs can form inactive oligomers via intermolecular cysteine disulfide bonds under oxidative conditions [107]. During cold stress, cytosolic thioredoxin h2 (Trx-h2) is rapidly activated and translocated into the nucleus, where it reduces these disulfide bonds, converting inactive CBF oligomers into active monomers. This regulatory process functions as a temperature-dependent molecular switch: under warm conditions, Trx-h2 is myristoylated and retained in the cytosol; however, cold exposure triggers Trx-h2 demyristoylation, which exposes its nuclear localization signal (NLS) and thereby promotes nuclear import. Phenotypically, Trx-h2-deficient plants exhibit decreased cold tolerance, while expression of a constitutively nuclear Trx-h2 variant enhances freezing resistance. Whether this Trx-h2–based redox switch is conserved in perennials remains unknown. These findings highlight the critical role of redox signaling in fine-tuning CBF activity [107].

#### 2.4.4. Chromatin Remodeling and Cold Memory

Cold acclimation is also regulated by chromatin-level modifications. During prolonged cold exposure (e.g., vernalization), epigenetic marks including H3K27me3 are deposited at loci such as *FLC*, leading to stably repression of flowering inhibitors [108]. Under acute cold stress, recent genome-wide chromatin studies have revealed dynamic changes in histone acetylation and methylation at *CBF* target loci [95]. Additionally, non-coding RNAs (ncRNAs), such as *COOLAIR* and *COLDAIR*, have been shown to contribute to the transcriptional regulation of cold-responsive genes under acute cold [109,110,111]. These findings suggest that cold memory, initially characterized in the regulation of flowering time, may also exert a broader regulatory role in the plasticity of stress responses.

#### 2.4.5. Chloroplast Retrograde Signaling and the Malate Valve

In parallel with chromatin-level control, organelle-to-nucleus communication couples cellular energy/redox status to nuclear programs. During cold nights or temperature downshifts, CO_2_ fixation and NADPH consumption decline, predisposing PSI to acceptor-side limitation. The chloroplast malate valve indirectly exports excess reducing equivalents via stromal NAD(P)-MDH isoforms and envelope dicarboxylate transporters, thereby regenerating NADP^+^ to protect PSI and convey redox status to the nucleus for transcriptional adjustment [112,113]. This valve is dual-gated: NADP-MDH activity must be constrained at night to avoid futile cycling with the oxidative pentose phosphate pathway, helping stabilize carbon flux and redox partitioning under extended dark or cold-night conditions [113]. Recent work also elevates the role of stromal NAD(H) in the light; by routing reducing power through enzymes such as PGDH3, the chloroplast can bolster the NADH pool to drive NAD-MDH and the malate valve, sustaining PSI electron-acceptor capacity and stress robustness under low-temperature photochemical constraints [113].

Concurrently, chloroplast-derived H_2_O_2_ operates as a rapid retrograde messenger: high-resolution imaging demonstrates photosynthesis-dependent H_2_O_2_ transfer from chloroplasts to nuclei on physiological timescales, where it modulates nuclear gene expression and coordinates early stress programs [114,115]. Integrating metabolite-based retrograde signals (malate/OAA shuttling) with redox (H_2_O_2_)-based cues refines the cold-responsive transcriptome—inducing antioxidant defenses, osmoprotectant synthesis, and membrane remodeling—to mitigate photooxidative damage in cold environments [115,116,117]. It is still unclear if malate-valve retrograde signaling is universally deployed across taxa. Collectively, these mechanisms highlight how the malate valve and chloroplast redox signaling provide a metabolism-to-transcription axis that is especially pertinent to cold-night acclimation.

#### 2.4.6. Physiological and Metabolic Adjustments

Cold-triggered signaling converges on physiological and metabolic adjustments that keep membranes functional and preserve photosynthesis. Plants raise the unsaturated/saturated lipid ratio via plastidic/ER desaturases and tune MGDG/DGDG and related lipid classes to stabilize thylakoids and protein-lipid microdomains at low temperature [118,119]. In parallel, COR programs induce LEA/dehydrins, antifreeze proteins, and molecular chaperones (HSPs) to maintain proteostasis. Carbohydrate partitioning shifts toward sucrose and raffinose-family oligosaccharides (RFOs) via GOLS/RS, with nocturnal starch remobilization supporting energy supply; compatible solutes such as proline and trehalose add osmotic and radical-buffering capacity [120].

Low temperature promotes acceptor-side limitation and ROS build-up; the ascorbate-glutathione (AsA-GSH) cycle, together with SOD, CAT, peroxiredoxins and thioredoxins, constrains oxidative damage, whereas the glyoxalase pathway (Gly I/II, often coupled with D-lactate dehydrogenase) detoxifies methylglyoxal (MG) to limit glycation and shape MG-dependent signaling. In concert, these carbonyl- and redox-processing modules coordinate the amplitude and range of H_2_O_2_ and MG signals to refine nuclear programs during cold acclimation [23,33,121,122]. To avoid over-reduction, the chloroplast malate valve exports excess reducing equivalents via NAD(P)-MDH and dicarboxylate transporters, sustaining NADP^+^/NADPH balance and integrating with H_2_O_2_-based retrograde signals; mitochondrial AOX provides an overflow electron sink. Collectively, these adjustments reduce electrolyte leakage and sustain Fv/Fm, NPQ and organ performance during recurrent cold exposure [123].

### 2.5. Specific Regulatory Modules Under Combined Stresses

**Figure 5:**
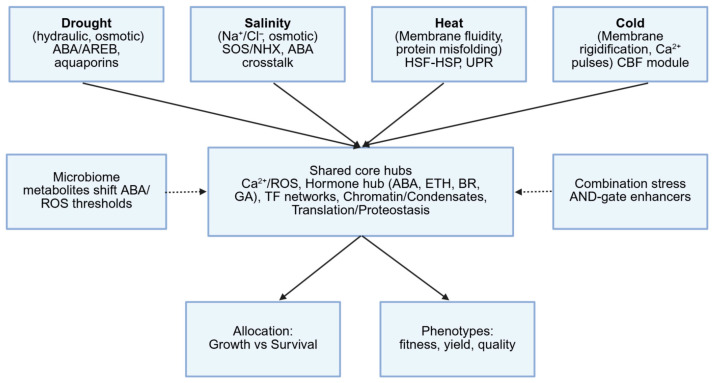
Conceptual framework linking single- and combined-stress responses. Drought, salinity, heat and cold converge on shared hubs (Ca^2+^/ROS, hormone crosstalk, TF networks, chromatin/condensates, translation/proteostasis) that allocate outputs to growth vs. survival and phenotypes. Dashed elements mark testable features relevant to combinations-AND-gate enhancers and microbiome-shifted ABA/ROS thresholds-which help explain the drought + heat conflicts (e.g., ABA-driven closure vs. heat-driven cooling) and salinity + cold co-regulation (CBF regulon with ion homeostasis and antioxidant programs). This schematic provides the backdrop for Section 2.5.1 and Section 2.5.2, where we detail ELF6-mediated DH acclimation, glutathione dependence under cold + osmotic stress, and the MsMYB12→MsFLS13 flavonoid module in alfalfa.

In natural environments, plants frequently encounter multiple stresses simultaneously, and their responses are not simple overlays of single-stress programs but distinct regulatory modules. While shared hubs (e.g., ABA and ROS) provide broad cross-protection, each stress combination can activate dedicated circuitry tuned to that specific scenario. Below, we highlight two common combinations—drought–heat and salinity–cold—to illustrate how plants orchestrate specialized networks beyond single-stress pathways. These interconnections are summarized in a minimal conceptual schematic (Figure 5).

#### 2.5.1. Drought–Heat Combination

Drought and heat often co-occur (e.g., heatwaves during water deficit), imposing concurrent dehydration, overheating, and oxidative stress. Plants under combined drought–heat display unique transcriptomic signatures and regulatory interactions that differ markedly from either stress alone [32]. Meta-analyses indicate that hundreds of transcripts—including many HSF, MYB, and ERF transcription factors—are specifically induced by combined heat–drought (and other heat-related combinations), with expression patterns absent under single stresses [32]. In grapevine, most genes induced by the combined treatment (e.g., particular heat-shock proteins, MAP kinases, and chromatin modifiers) are unique to the combination, rather than shared with drought-only or heat-only responses [124]. These observations indicate that drought + heat activates novel regulators and pathways, rather than a simple sum of single-stress responses. This drought–heat conflict and its resolution via hormonal crosstalk are highlighted in the framework (Figure 5).

A key physiological conflict is that drought promotes ABA-driven stomatal closure to conserve water, whereas heat favors stomatal opening to enhance leaf cooling. Under combined stress, hormonal crosstalk resolves this conflict: elevated ABA under water deficit suppresses auxin-driven thermomorphogenic growth, prioritizing water conservation over leaf expansion [125,126]. Consistently, field and lab studies note that plants modulate these “gas-and-brake” signals (closure for water saving vs. opening for cooling) to optimize survival under combined stress [127]. At the regulatory level, specific “master” genes can serve as hubs of combined-stress tolerance. For example, in *Arabidopsis*, the chromatin regulator EARLY FLOWERING 6 (ELF6) was identified as uniquely required for acclimation to simultaneous high temperature and drought, underscoring that certain transcriptional regulators are dedicated to combined-stress contexts [128]. Together, these findings show that drought–heat elicits specialized modules—integrating unique TF combinations, hormonal crosstalk, and epigenetic factors—that enable plants to cope more effectively with compounded stress than with isolated pathways.

#### 2.5.2. Salinity–Cold Combination

High salinity frequently coincides with cold (e.g., saline soils in cold seasons). Both impose osmotic strain, but salinity adds ionic toxicity, while cold reduces membrane fluidity and can cause freezing injury. Under salinity–cold, plants activate integrative defense modules to manage water deficit, ion homeostasis, and low-temperature injury simultaneously. Many osmoprotective genes classically induced by drought or cold (e.g., RD29A/RD29B and other dehydrins under the DREB/CBF regulon) are also induced by high salinity [129], reflecting a convergent protective strategy in which compatible solutes and LEA proteins support both freezing tolerance and salt-osmotic tolerance.

Beyond this overlap, the combination often triggers additional, unique responses. Plants under simultaneous salt and cold exhibit a heightened requirement for antioxidant capacity and hormonal coordination. For instance, glutathione-deficient *Arabidopsis* mutants are especially sensitive to combined cold + osmotic stress, with aberrant induction of stress-responsive genes-indicating that robust ROS scavenging and the interplay among ABA, ethylene, auxin, and brassinosteroid signaling are crucial for combined-stress tolerance [126]. Recent multi-omics work in alfalfa exposed to cold + saline-alkaline conditions uncovered a specialized R2R3-MYB module: MsMYB12 upregulates MsFLS13 (a flavonol synthase), boosting flavonoid biosynthesis and antioxidant capacity specifically under the combined stress; overexpressing MsFLS13 enhances tolerance to the salinity–cold combination [130]. Accordingly, flavonoid antioxidants emerge as key protective compounds in dual stress, mitigating oxidative damage and stabilizing membranes [130]. Combined salinity–cold can also elevate jasmonic acid and sugar levels in tolerant genotypes, pointing to metabolic reprogramming (e.g., higher osmolyte and energy reserves) as an additional layer of adjustment. In sum, salinity–cold prompts plants to deploy combination-specific regulatory networks that integrate the CBF regulon, salt-specific ion homeostasis, and enhanced antioxidant/metabolic programs, collectively enabling survival in cold, saline environments.

### 2.6. Heavy Metal (HM) Stress Responses

Heavy metal pollution, particularly from cadmium (Cd), arsenic (As), lead (Pb), and mercury (Hg), has emerged as a critical environmental issue threatening global agroecological security and human health [131]. These toxic elements severely inhibit plant growth and development by disrupting cellular structures, interfering with enzymatic activities, inducing oxidative stress, and competing with essential nutrients for uptake sites [132]. To counteract such stresses, plants have evolved a multilayered and highly coordinated defense system, encompassing external exclusion, intracellular sequestration and compartmentalization, antioxidant defense, signal transduction, and reprogramming of gene expression [132,133]. This system effectively detoxifies toxic ions and maintains basic physiological processes. This section systematically elaborates on the key physiological and molecular mechanisms underlying plant responses to heavy metal stress.

#### 2.6.1. Root Avoidance Responses and Rhizospheric Processes

To minimize heavy metal uptake at the source, plants employ an ‘avoidance’ strategy by modulating root architecture and the rhizosphere microenvironment. In terms of root morphology, plants remodel their root systems via the ‘RBOH-ROS-Auxin’ signaling pathway, promoting primary root deviation from contaminated zones while stimulating lateral root growth toward uncontaminated areas [134]. At the rhizosphere level, organic acids (e.g., citric acid, malic acid), phenolic compounds, and mucilage secreted by roots can immobilize heavy metals via chelation, precipitation, or altering rhizosphere pH, thereby reducing their bioavailability [135,136]. Recent studies indicate that, specific plant growth-promoting rhizobacteria (PGPR; e.g., *Azospirillum brasilense*) can form mutualistic associations with plants, synergistically enhancing heavy metal detoxification efficiency and significantly reducing metal accumulation in plant tissues [137,138]. These rhizospheric processes are governed by precise regulation of specialized transport proteins and hormonal signaling networks, representing a critical initial mechanism by which plants enact heavy metal ‘avoidance’ strategies.

#### 2.6.2. Cell Wall Fixation, Intracellular Chelation, and Vacuolar Sequestration

The cell wall serves as the first barrier against heavy metal entry into plant cells. Polysaccharide components like cellulose and pectin can retain substantial amounts of metal ions through adsorption and co-precipitation [139]. Upon entry into the cytoplasm, plants primarily rely on phytochelatins (PCs) and metallothioneins (MTs) for intracellular chelation and detoxification. PCs are synthesized through the polymerization of glutathione (GSH) catalyzed by phytochelatin synthase (PCS) and exhibit high affinity for Cd and As [140]. MTs effectively bind metals such as Cu, Zn, and Cd via their cysteine residues [141]. Currently, different subtypes of PCS and MT genes from various species (e.g., *BnPCS1* in *Brassica napus*, *OsMT-I-Id* in rice) have been cloned and functionally validated [142,143], providing valuable genetic resources for improving plant tolerance to heavy metals.

During vacuolar sequestration, the transport of PC-metal complexes into the vacuole depends not only on ABC transporters (e.g., AtABCC1/2 in *Arabidopsis*) and heavy metal ATPases (HMAs, e.g., AtHMA3) but also requires the involvement of the coat protein Sec24C [144,145,146]. Sec24C can directly interact with AtABCC1/2, facilitating the localization of these complexes to the tonoplast via a Golgi-independent pathway involving the endoplasmic reticulum-prevacuolar compartment-tonoplast (ER-PVC-TP) route. Disruption of this pathway results in the retention of AtABCC1/2 in the ER, significantly impairing plant tolerance to Cd and As [145]. In crop improvement applications, loss-of-function of *OsHMA3* in rice leads to Cd accumulation in shoots, whereas its functional enhanced allele can reduce grain Cd content [147]. Furthermore, downregulating the expression of *TaHMA3* and *TaNramp1* in wheat via application of a *Chlamydomonas reinhardtii* preparation reduced root Cd content by 76.80% [148]. These findings offer new targets and technical pathways for low-Cd crop breeding and heavy metal pollution control.

#### 2.6.3. Oxidative Stress and Activation of Antioxidant Defense

Heavy metal stress directly or indirectly triggers excessive accumulation of reactive oxygen species (ROS), leading to oxidative damage in cellular components. Research shows that GSH functions not only as a precursor for PC synthesis but also as a crucial antioxidant molecule, eliminating peroxides through the ascorbate-glutathione (AsA-GSH) cycle [149,150]. Additionally, the recognition of toxic heavy metal ions by specific receptors on the root cell membrane can activate RBOH-mediated ROS (e.g., H_2_O_2_) production, which in turn initiates intracellular oscillatory Ca^2+^ signals and MAPK cascade reactions, thereby regulating the expression of antioxidant defense genes and establishing systemic resistance [151,152]. Recent studies have shown that under heavy metal stress, ROS generated by plant roots act as a ‘distress signal’ to facilitate the recruitment of plant growth-promoting rhizobacteria (PGPR), such as *Bacillus* sp. PGP5 and *Pantoea* sp. PGP6, which synthesize indole-3-acetic acid (IAA) [153]. In turn, IAA mitigates excessive ROS accumulation in roots, establishing an ‘ROS-IAA’ negative feedback loop. Additionally, IAA can modulate the expression of DNA methylation-related genes in plant roots, thereby refining the cross-kingdom ‘ROS-IAA-methylation’ signaling pathway and establishing a long-term stress resistance mechanism [153].

#### 2.6.4. Hormonal Signaling and Epigenetic Regulation

At the level of hormonal signaling regulation, beyond the traditional salicylic acid (SA), jasmonic acid (JA), and abscisic acid (ABA), the mechanisms of gibberellin (GA) and melatonin (Mel) are gradually elucidated. Recent studies have shown that under the combined stress of nutrient deficiency and heavy metals, GA can degrade the growth repressor DELLA proteins (RGL2/RGA) through the GID1 receptor-mediated ATG8-dependent autophagic pathway, relieve its inhibition on seed germination and seedling growth, and provide a new pathway for the regulation of growth-stress resistance balance under stress [154]. Melatonin (Mel), on the other hand, targets glucose-6-phosphate dehydrogenase (G6PD), shifts glucose metabolism from glycolysis to the pentose phosphate pathway, inhibits lead (Pb)-induced histone lactylation (H3K18la) and mitochondrial fragmentation, and alleviates pyroptotic damage [155,156].

In the field of epigenetic regulation, new discoveries are emerging continuously. In addition to the classic DNA methylation and H3K4me3 histone modification, histone lactylation (H3K18la) and long non-coding RNA (LncRNA) have also become research hotspots [157,158]. Existing studies indicate that histone lactylation (H3K18la), as a key nexus between metabolism and epigenetic regulation, is activated under Pb stress through the accumulation of lactate—a glycolysis byproduct—and regulates the expression of the mitochondrial homeostasis-related gene *DRP1*, participating in toxic responses [159]. Under Cd stress, the birch *LncRNA2705.1* upregulates the expression of its target gene *HSP18.1*, while *LncRNA11415.1* indirectly activates the LLDA gene through downregulation of its expression, both significantly enhancing Cd resistance [159].

#### 2.6.5. Directed Allocation and Stress Memory

The directed transport and allocation of heavy metals within plants are coordinately controlled by multiple transporter families, including HMA, ZIP, NRAMP, and YSL, forming a sophisticated regulatory network. For instance, among HMA family members, OsHMA2 mediates the translocation of Zn/Cd from roots to shoots, whereas OsHMA3 facilitates vacuolar sequestration of Cd [146,160]. The NRAMP family (e.g., OsNRAMP5) is involved in root absorption of Mn and Cd. The YSL family is primarily responsible for phloem transport of metal-nicotianamine complexes and regulates redistribution between young and senescing tissues [161].

Emerging studies reveal that exposure to heavy metals (e.g., Cd and Cr) can induce siRNA-mediated transgenerational memory in rice and *Arabidopsis* [162,163]. The core mechanism involves persistent hypomethylation at CHG sequences of heavy metal-responsive genes and transposable elements (e.g., Tos17), a process associated with the downregulation of the maintenance methyltransferase CMT3 and upregulation of demethylase activity. This epigenetic memory leads to increased expression of heavy metal transporters (e.g., OsHMA), enhancing vacuolar sequestration or efflux [163]. This not only confers stronger heavy metal tolerance to the progeny but can also generate broad-spectrum resistance to cross-stresses like salt stress and MMS [164], providing new perspectives for understanding plant adaptive evolution and stress-resistant breeding.

## 3. Future Prospects and Conclusions

Recent research has significantly advanced our understanding of how plants perceive and respond to abiotic stresses, uncovering a highly integrated, multilayered network of regulatory mechanisms. While each stress—drought, salinity, heat, cold, and heavy metal—triggers distinct signaling cascades, several core features are shared. These include calcium and reactive oxygen species (ROS) signaling, hormonal regulation (most notably abscisic acid, alongside ethylene, brassinosteroids, and gibberellins), and transcriptional reprogramming driven by key regulators. Additionally, plants employ extensive post-transcriptional and post-translational mechanisms to fine-tune their responses. High-impact studies have further emphasized the importance of subcellular compartmentalization (e.g., vacuolar and chloroplast-based signaling), 3D chromatin architecture, and biomolecular condensates, underscoring the remarkable complexity of plant stress adaptation. Together, these observations can be organized into a concise sensor-integration-allocation model: primary triggers feed into Ca^2+^/ROS and hormone hubs (sensor), tissue- and history-dependent modules (chromatin, condensates) compute context (integration), and translational/proteostatic controls execute resource partitioning between survival and growth (allocation). This model explains why conserved modules recur yet yield divergent outcomes across tissues and stress histories.

A persistent challenge in stress biology is achieving stress tolerance without compromising growth. Several recent breakthroughs have offered promising solutions: for instance, overexpression of the vascular-specific brassinosteroid receptor BRL3 enhances drought tolerance without growth penalties [29], while the SALTY protein preserves translation under high salinity by preventing ribosome stalling [61]. These findings illustrate viable strategies to uncouple defense responses from growth repression—an essential goal for sustainable agriculture.

Despite this progress, numerous questions remain unresolved. The molecular identity of primary stress sensors—whether membrane-bound receptors, metabolic intermediates, or conformationally sensitive macromolecules—remains unclear in many contexts. The plant microbiome, increasingly recognized as a modulator of hormonal and stress responses, introduces an additional layer of complexity requiring further exploration [19]. Moreover, in natural environments, stresses often occur in combination: recent studies demonstrate that plant responses to combined stresses (e.g., drought plus heat) are not merely additive but involve unique regulatory programs [31,32,165]. Specific genes are induced exclusively under combined stress conditions, suggesting the existence of specialized signaling hubs. Conceptual frameworks such as Mittler’s ‘stress matrix’ are being continuously refined to better map these interactive effects and guide crop engineering under real-world conditions [32] (Figure 5). We hypothesize that combination-specific genes are governed by AND-gate enhancers that require dual upstream inputs, while microbiome-derived metabolites shift ABA/ROS thresholds, thereby reprogramming these gates. Falsifiable tests include CRISPR perturbation of predicted dual-input motifs and metabolite complementation to rescue gate activation under combined stress.

Translational applications are now emerging from these insights. Genome editing tools, particularly CRISPR/Cas systems, enable precise manipulation of stress-regulatory genes: CRISPR-mediated activation of *AREB1* confers drought tolerance in *Arabidopsis* with minimal growth cost [27], while CRISPR knockout of negative regulators (e.g., *OsPP2C68*) enhances ABA sensitivity and improves both drought and salinity tolerance in rice [166]. Tissue-specific manipulation of signaling components, such as root-specific aquaporins or stress-responsive receptors, offers promising strategies to boost resilience while minimizing trade-offs. Nevertheless, care must be taken to avoid fitness penalties, highlighting the importance of context-dependent or inducible regulatory strategies. Accordingly, we propose a two-step pipeline: (i) map where/when/how strongly to intervene using tissue-resolved omics and inducible reporters; (ii) deploy cell type-specific CRISPRa/CRISPRi edits at validated hubs, benchmarked under field-mimetic fluctuating regimes.

In conclusion, plant responses to abiotic stress are governed by a dynamic and interconnected regulatory network. This review has summarized recent advances in understanding signal perception, transduction, and response integration, spanning hydraulic cues, multisensor thermosensing, redox regulation, systemic ROS signaling, and chromatin modification-mediated stress memory. These insights not only deepen our understanding of plant biology but also inform innovative strategies for crop improvement. Beyond synthesis, this review advances the field by (i) articulating the sensor–integration–allocation framework that reconciles divergent results; (ii) deriving testable predictions for combination stress and microbiome modulation; and (iii) formalizing engineering rules for tissue-restricted and process-level interventions. These propositions are immediately actionable with standardized multi-omics, live-cell imaging of condensates, and synthetic biology toolkits, providing an explanatory and predictive basis for designing climate-resilient crops.

## Data Availability

No new data were created or analyzed in this study. Data sharing is not applicable to this article.

## Abbreviations

The following abbreviations are used in this manuscript:

ABA	Abscisic Acid
Ca^2+^	Calcium ions
ROS	Reactive oxygen species
SA	Salicylic Acid
JAs	Jasmonates
GAs	Gibberellins
BRs	Brassinosteroids
CRISPRa	CRISPR activation
SOS	Salt Overly Sensitive
CIPK24	CBL-Interacting Protein Kinase 24
CBL4	Calcineurin B-like Protein 4
HKT1	high-affinity K^+^ transporters
RLK	receptor-like kinase
GIPC	glycosyl inositol phosphorylceramides
NF-Y	nuclear factor Y
TCA	tricarboxylic acid
GABA	γ-aminobutyric acid
HSR	heat shock response
HSP	heat shock protein
HSF	heat shock transcription factor
RBP	RNA-binding protein
phyB	phytochrome B
PIF4	PHYTOCHROME INTERACTING FACTOR 4
ICE1	Inducer of CBF Expression 1
COR	cold-responsive
HOS1	High expression of osmotically responsive genes 1
CAMKII	calmodulin kinase II
Trx-h2	thioredoxin h2
NLS	nuclear localization signal
ncRNA	non-coding RNA
HM	Heavy Metal
Cd	Cadmium
As	Arsenic
Pb	Lead
Hg	Mercury
PGPR	Plant Growth-Promoting Rhizobacteria
PCs	Phytochelatins
MTs	Metallothioneins
GSH	Glutathione
PCS	Phytochelatin Synthase
ABC transporters	ATP-Binding Cassette transporters
HMAs	Heavy Metal ATPases
ER	Endoplasmic Reticulum
PVC	PreVacuolar Compartment
TP	Tonoplast
Mel	Melatonin
IAA	Indole-3-Acetic Acid
LncRNA	Long non-coding RNA
siRNA	Small interfering RNA
